# Pre-clinical imaging of transgenic mouse models of neuroblastoma using a dedicated 3-element solenoid coil on a clinical 3T platform

**DOI:** 10.1038/bjc.2017.251

**Published:** 2017-08-08

**Authors:** Gilberto S Almeida, Rafal Panek, Albert Hallsworth, Hannah Webber, Efthymia Papaevangelou, Jessica KR Boult, Yann Jamin, Louis Chesler, Simon P Robinson

**Affiliations:** 1 Division of Radiotherapy & Imaging, The Institute of Cancer Research, 15 Cotswold Road, Sutton, Surrey SM2 5NG, UK; 2 Division of Clinical Studies, The Institute of Cancer Research, 15 Cotswold Road, Sutton, Surrey, SM2 5NG, UK

**Keywords:** MRI, pre-clinical, neuroblastoma, clinical platform, murine model, cancer imaging

## Abstract

**Background:**

The use of clinical MRI scanners to conduct pre-clinical research facilitates comparisons with clinical studies. Here the utility and sensitivity of anatomical and functional MRI data/biomarkers acquired from transgenic mouse models of neuroblastoma using a dedicated radiofrequency (RF) coil on a clinical 3T scanner was evaluated.

**Methods:**

Multiparametric MRI of transgenic mice bearing abdominal neuroblastomas was performed at 3T, and data cross-referenced to that acquired from the same mice on a pre-clinical 7T MRI system. *T*_2_-weighted imaging, quantitation of the native longitudinal relaxation time (*T*_1_) and the transverse relaxation rate (*R*_2_*), and dynamic contrast-enhanced (DCE)-MRI, was used to assess tumour volume, phenotype and response to cyclophosphamide or cabozantinib.

**Results:**

Excellent *T*_2_-weighted image contrast enabled clear tumour delineation at 3T. Significant correlations of tumour volume (*R*=0.98, *P*<0.0001) and *R*_2_* (*R*=0.87, *P*<0.002) measured at 3 and 7T were established. Mice with neuroblastomas harbouring the anaplastic lymphoma kinase mutation exhibited a significantly slower *R*_2_* (*P*<0.001), consistent with impaired tumour perfusion. DCE-MRI was performed simultaneously on three transgenic mice, yielding estimates of *K*^trans^ for each tumour (median *K*^trans^ values of 0.202, 0.168 and 0.114 min^−1^). Cyclophosphamide elicited a significant reduction in both tumour burden (*P*<0.002) and native *T*_1_ (*P*<0.01), whereas cabozantinib induced significant (*P*<0.01) tumour growth delay.

**Conclusions:**

Simultaneous multiparametric MRI of multiple tumour-bearing animals using this coil arrangement at 3T can provide high efficiency/throughput for both phenotypic characterisation and evaluation of novel therapeutics, and facilitate the introduction of functional MRI biomarkers into aligned imaging-embedded clinical trials.

High field (>4.7T) small animal dedicated MRI systems that provide high resolution and signal to noise ratios (SNR) have and continue to be the preferred platform for pre-clinical imaging investigations. The physical contrast characteristics and imaging performance of such pre-clinical scanners differs substantially from clinical MRI systems. The use of clinical scanners to conduct pre-clinical research is attractive, as it facilitates a more direct comparison with clinical studies, the application and development of clinically applicable imaging protocols, matching of field strength-related mechanisms such as relaxation, and provides evidence to support the clinical relevance of functional MRI data/biomarkers (Brockmann *et al*, 2007; Chen *et al*, 2007; Inderbitzin *et al*, 2007; Linn *et al*, 2007; Bilgen, 2013). This approach also benefits from the hardware and software advances continuously being made on modern clinical MRI systems, such as parallel transmit and receive technology, motion and contrast tracking, k-space under-sampling and view-sharing strategies (Ullman *et al*, 2004; Wech *et al*, 2012; Jerome *et al*, 2016). Additionally, the generous homogeneous field region of clinical scanners allows for high quality, whole-body pre-clinical imaging, and the potential for simultaneous data acquisition from several subjects during the same scanning session (Dazai *et al*, 2004; Nieman *et al*, 2007). Nuclear relaxation times are also often better differentiated at relatively lower field strengths (de Graaf *et al*, 2006), leading to superior contrast between imaged soft tissues, crucial for reliable organ and pathology delineation.

Most clinical scanners operate at between 1 and 3T, having lower SNR levels than pre-clinical high field systems, and hence reduced image quality if standard clinical imaging coils are used. This is exacerbated particularly when imaging the small fields-of-view necessary while using rodents, but with a high enough resolution to be able to accurately resolve lesions and acquire meaningful functional data. One approach of increasing SNR is to use small dedicated radiofrequency (RF) receiver coils. Such coils can be designed to fit closely to the object of interest, giving a better coupling between the object and coil (high filling factor), a stronger signal and thus improved image quality.

In pre-clinical cancer research performed *in vivo*, transgenic mouse models, in which tumours are driven by expression of the target gene of interest and arise spontaneously within the native tissue of origin, are being increasingly exploited (de Jong *et al*, 2014). These models more faithfully emulate human tumour growth, tumour-host stromal interactions and vasculature, metastatic potential and therapeutic response *in vivo*. Such models demand non-invasive methods to longitudinally and accurately assess the progression and treatment response of tumours that typically arise within deep-seated anatomical locations.

The purpose of this study was to evaluate the utility and sensitivity of a non-bespoke high resolution RF coil for use on a clinical 3T scanner for the acquisition of anatomical and functional MRI data/biomarkers from transgenic mouse models of neuroblastoma *in vivo*.

## Materials and methods

All procedures involving animals were performed in accordance with the local ethical review panel, the UK Home Office Animals (Scientific Procedures) Act 1986, the United Kingdom National Cancer Research Institute guidelines for the welfare of animals in cancer research and the ARRIVE guidelines (Workman *et al*, 2010; Kilkenny *et al*, 2010).

### *T*_1_ phantom

To assess the linearity of *T*_1_ measurements on both the 3 and 7T platforms, phantoms with increasing concentrations of gadolinium (from 30 to 500 nM) were imaged in both scanners at ambient temperature. These solutions were transferred into 5 mm NMR tubes, which in turn were placed in 50 ml falcon tubes and restrained using dental paste to limit motion artefacts. The phantoms were scanned four times on each scanner, and the average value used to assess the *T*_1_ field dependence.

### Transgenic mouse models and drug treatment

The aberrant expression of the transcription factor *MYCN* is a potent oncogenic stimulus in cancer. *MYCN* expression correlates with an aggressive tumour phenotype, enhanced tumour angiogenesis and poor clinical prognosis in neuroblastoma (Maris, 2010). Many paediatric cancers arise through the aberrant expression of just a few driver genes, and are thus amenable to transgenic mouse modelling approaches (Chesler and Weiss, 2011). For example, targeted overexpression of *MYCN* to the neural crest under the control of the tyrosine hydroxylase promoter resulted in the Th-*MYCN* transgenic mouse model in which abdominal tumours spontaneously develop that faithfully replicate the disease biology of high-risk neuroblastoma (Weiss *et al*, 1997). More recently, the identification of mutations in the anaplastic lymphoma kinase (*ALK*) gene in neuroblastoma, and its close association with *MYCN* amplification, stimulated the development of a novel double transgenic mouse model for *ALK* and *MYCN* (Berry *et al*, 2012). In both the Th-*MYCN* and Th-*ALK*^*F1174L*^/Th-*MYCN* transgenic mice, solid tumours spontaneously arise within the retroperitoneum in peri-renal and para-spinal abdominal regions, consistent with the typical clinical distribution and radiological presentation of human neuroblastoma.

In this study, male and female tumour-bearing Th-*MYCN* and Th-*ALK*^*F1174L*^/Th-*MYCN* mice, initially identified by palpation, underwent MRI at around 50 and 30 days old, respectively (Berry *et al*, 2012; Rasmuson *et al*, 2012). Their genotype was confirmed by analysing DNA from the tail using real-time quantitative reverse transcription polymerase chain reaction.

### MRI data acquisition

#### Study protocol

Twenty-three transgenic mice (Th-*MYCN n*=14, Th-*ALK*^*F1174L*^/Th-*MYCN n*=9) underwent imaging on the 3T platform. Of these, 10 mice (Th-*MYCN n*=7, Th-*ALK*^*F1174L*^/Th-*MYCN n*=3) were scanned at both 3 and 7T within 24 h of each other in order to compare tumour volume determination and quantification of tumour *R*_2_* at 3 and 7T. Dynamic contrast-enhanced (DCE)-MRI was performed simultaneously on three Th-*MYCN* mice at 3T. To evaluate the 3T platform for imaging-embedded intervention trials, anatomical and functional (native *T*_1_ and *R*_2_*) MRI measurements were acquired from Th-*MYCN* tumours prior to and 24 h post-treatment with either saline (control, *n*=4) or a single 25 mg kg^−1^ i.p. dose of the DNA alkylating agent cyclophosphamide (CPM, *n*=5), or prior to and 48 h post treatment with a daily oral dose of either water (control, *n*=*4*) or 30 mg kg^−1^ of the multi-kinase inhibitor cabozantinib (CBZ, *n*=5) (Yakes *et al*, 2011).

#### 3T data acquisition and analysis

**Figure 1:**
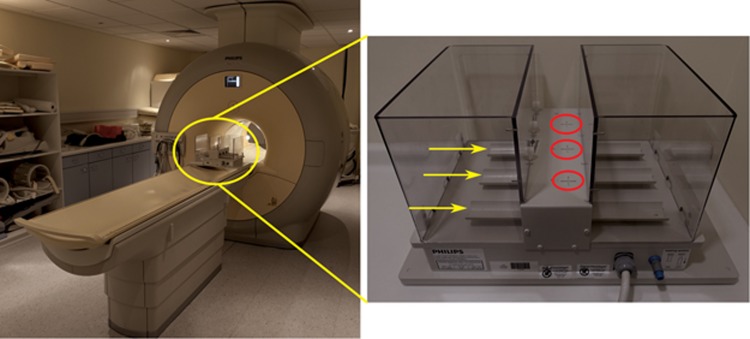
**‘Mouse hotel’ coil and the 3T clinical scanner.** Photographs of the coil arrangement on the 3T scanner, and the high resolution 3-channel/3-animal MR RF coil. The yellow arrows indicate the three individual mouse beds and the red circles the centre of each element of the coil. Positioning of the coil in the scanner as shown means the mice are perpendicular to the *B*_0_ field.

MRI data was acquired on a clinical scanner (Philips Achieva, Best, Netherlands) using a dedicated high-resolution RF coil (‘Mouse Hotel’, Philips, Best, Netherlands) (Figure 1). The coil consists of three 40 mm inner diameter solenoids, with the long axis oriented perpendicular to the magnet *B*_0_ field. This coil arrangement enables the simultaneous acquisition of MRI data from up to three animals using independent receiver channels. The 3T imaging protocol, which included *T*_2_-weighted, *T*_1_ and *R*_2_* measurements, was tested and optimised using phantoms prior to the *in vivo* study. A compromise between SNR, spatial and temporal resolution was prioritised over a direct parameter match to pre-clinical 7T protocols.

Anaesthesia was induced by intraperitoneal injection of midazolam (5 mg kg^−1^), fentanyl (0.05 mg kg^−1^), and medetomidine (0.5 mg kg^−1^) in sterile water. For 3T data acquisition, up to three anaesthetised mice were positioned supine with their abdomens in the centre of each individual element of the coil. Mouse core body temperature was maintained by a built-in heating system controlled within the ‘Mouse Hotel’ coil.

Initially, whole body anatomical *T*_2_-weighted images were acquired at 3T using a coronal orientation with full anatomical coverage of all three animals (TSE, 20 slices, slice thickness: 1 mm, FOV=250 × 200 mm, resolution=0.2 × 0.2 mm, ETL=14, TE/TR=80/3000 ms, NSA=1, TA=3 min 48 s). *T*_2_-weighted images were used to assess the extent of the disease and aid axial sequence planning, which was performed across the central part of the tumour. Proton density and *T*_1_-weighted images were then acquired using a 3D spoiled gradient echo sequence with variable flip angle (axial orientation, 7 slices, slice thickness: 1.5 mm, resolution=0.25 × 0.25 mm, FOV=200 × 85 mm, FA=3 and 16°, 10 dummy scans, TE/TR=2.3/7 ms, NSA=1, TA=27 s). Multigradient echo (MGE) images were acquired using a 2D gradient echo sequence with multiple echo times (axial orientation, 3 slices, slice thickness: 1.5 mm, resolution=0.3 × 0.3 mm, FOV=200 × 50 mm, FA=24°, TE=4.6, 11.5, 18.4, 25.3, 32.2 and 39.1 ms, TR=500 ms, NSA=1, TA=30 s). No fat suppression was employed.

For the acquisition of DCE-MRI data, a lateral tail vein of each mouse was cannulated with a 27G butterfly catheter connected to a 10 m long line of polyethylene tubing (BPTE-T10, 0.23″ i.d., 0.38 ″ o.d.), and attached through the available wave-guides to syringe pumps located in the scanner control room, thereby enabling the simultaneous remote intravenous administration of contrast agent to each mouse. DCE-MRI data were acquired using the 3D spoiled gradient echo sequence with 200 time points, a temporal resolution of 2.5 s and a total acquisition time of 8 mins 33 s. A bolus injection of 0.1 mmol kg^−1^ gadopentate dimeglumine (Gd-DTPA) was simultaneously administered to all three mice at a rate of 2 ml min^−1^, 30 s after the start of the dynamic acquisition. The total 3T protocol scanning time was less than 15 min.

Tumour volumes were extrapolated from manually drawn regions of interest (ROIs) on *T*_2_-weighted images for each tumour-containing slice, where clear delineation of tumour could be determined, using OsiriX Lite (Pixmeo). The *T*_1_-weighted images were analysed using dedicated software (MRIW, working under IDL, (d’Arcy *et al*, 2006)), and the ratio between the proton density (FA=3°) and *T*_1_-weighted (FA=16°) 3D spoiled gradient echo used to provide estimates of the native tumour spin-lattice relaxation time *T*_1_ (ms) (Fram *et al*, 1987). The MGE data were analysed using another dedicated software (ADEPT, working under IDL (Doran *et al*, 2012)), with tumour *R*_2_* (s^−1^) maps calculated by fitting a single exponential to the signal intensity echo time curve on a voxel-by-voxel basis using a Bayesian maximum *a posteriori* approach (Walker-Samuel *et al*, 2010). MRIW was used to fit the DCE-MRI data incorporating the Kety model (Tofts *et al*, 1999) and a literature-derived murine arterial input function (Benjaminsen *et al*, 2004), providing estimates of *K*^trans^ (min^−1^), the volume transfer constant between blood plasma and extracellular extravascular space, *V*_e_ (dimensionless volume fraction, and IAUGC_60_ (mM.s), the initial area under the gadolinium concentration curve from 0 to 60 s after injection of Gd-DTPA.

#### 7T data acquisition and analysis

Volumetric analysis and quantification of *T*_1_ and *R*_2_* of tumours arising within Th-*MYCN* and Th-*ALK*^*F1174L*^/Th-*MYCN* transgenic mice was performed on a 7T horizontal bore microimaging system (Bruker Instruments, Ettlingen, Germany) using a 3-cm birdcage volume coil, as previously described (Jamin *et al*, 2013, 2014).

#### Statistical analysis

Statistical analysis was performed using GraphPad Prism 6. The absolute values for tumour volume, and median values for *T*_1_ and *R*_2_* were used. Any significant difference within the same group was identified using Student’s two-tailed paired *t*-test, with a 5% level of significance. Any significant difference between groups after treatment was tested using an unpaired *t*-test. The linear correlation coefficient *R* was used to test correlation strength, direction and linear association between volumes and relaxation times measured at both field strengths.

## Results

Measurements from the *T*_1_ phantom for all Gd-DTPA concentrations at both field strengths are shown in Table 1. A highly significant correlation (*R*=0.99, *P*<0.0001) was found between the *T*_1_ values obtained from the 3T clinical scanner and the 7T pre-clinical scanner, with the median *T*_1_ values being higher at 7T.

**Figure 2:**
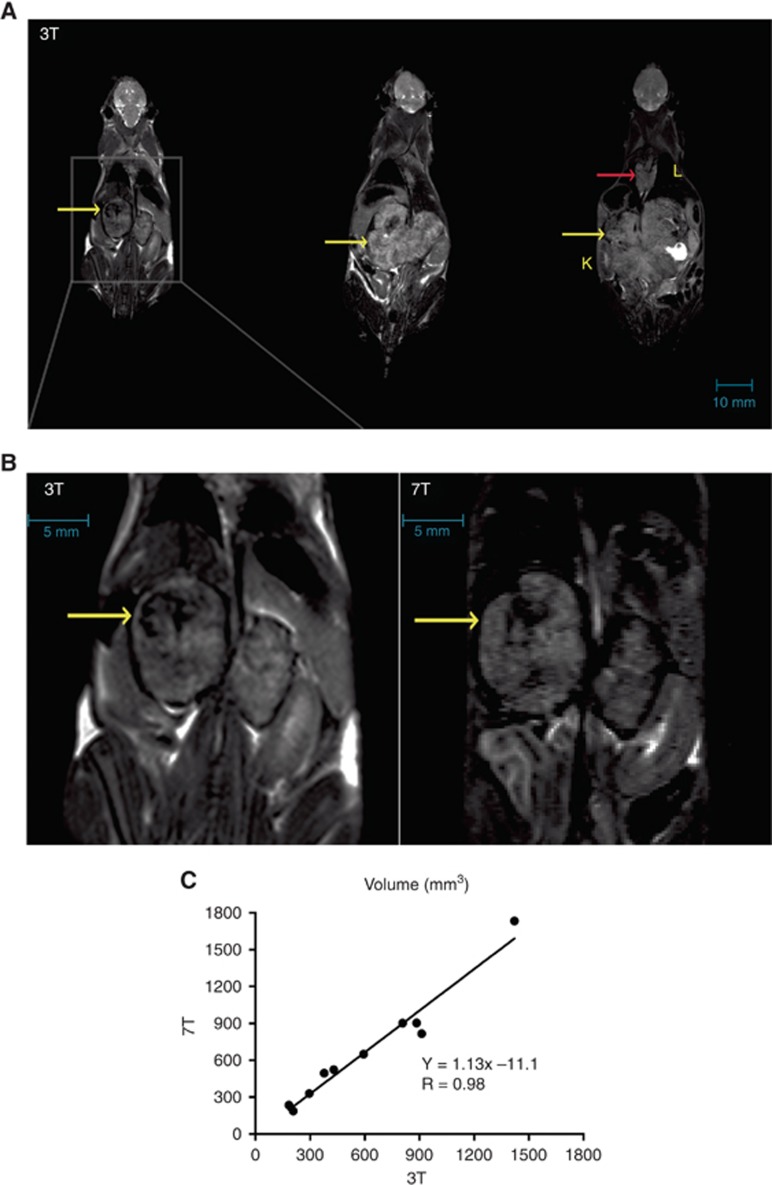
**Anatomical imaging at 3 and 7T.** (**A**) Anatomical *T*_2_-weighted coronal images acquired simultaneously from three Th-*MYCN* mice with abdominal neuroblastoma using the high resolution 3-channel/3-animal MR RF coil on the 3T clinical scanner, with a scale bar in blue. Each tumour is indicated by yellow arrows, with kidney (K) and lungs (L) also annotated. An additional thoracic lesion identified in one of the mice is indicated with a red arrow. (**B**) Expanded *T*_2_-weighted image of the abdomen of the first mouse in (**A**) acquired at 3T, and the corresponding *T*_2_-weighted image acquired from the same mouse at 7T, with corresponding scale bars in blue. (**C**) Linear regression analysis of the volumetric measurements determined from *T*_2_-weighted MRI of tumour-bearing transgenic mice imaged at 3 and 7T revealed a positive and highly significant correlation (*R*=0.98, *P*<0.0001).

Representative anatomical *T*_2_-weighted MR images of tumour-bearing mice acquired at 3 and 7T are displayed in Figure 2A and B, revealing large peri-renal masses within each mouse abdomen, and occasionally an additional thoracic tumour, consistent with the radiological presentation of neuroblastoma. The excellent image contrast yielded clear tumour delineation across contiguous slices that could be used for accurate volumetric analysis *in vivo*. No significant difference was found between volumetric measurements acquired at the different field strengths (mean tumour volume 819±153 mm^3^ at 3T *vs* 889±191 mm^3^ at 7T, *n*=10, *P*>0.05), and which were significantly correlated (*R*=0.98, *P*<0.0001, Figure 2C).

**Figure 3:**
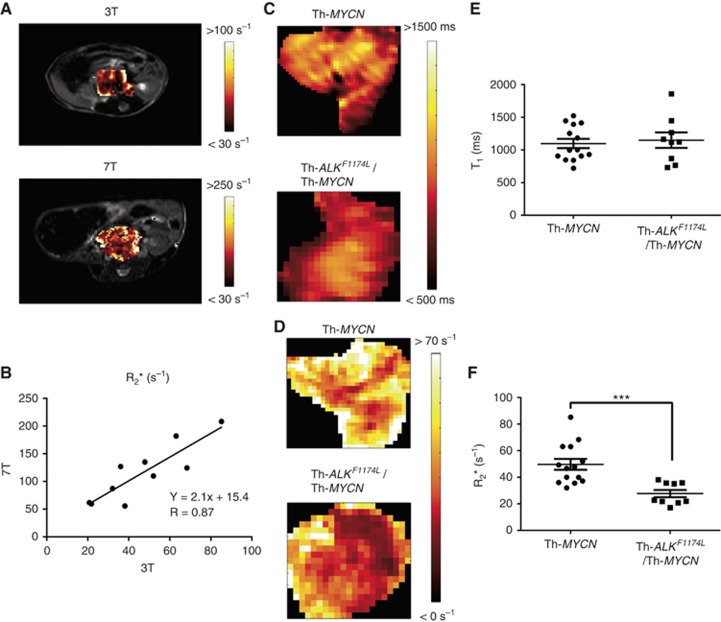
**Functional MRI of neuroblastomas at 3 and 7T.** (**A**) Parametric *R*_2_* maps acquired at 3 and 7T from the same Th-*MYCN* transgenic mouse bearing an abdominal neuroblastoma. Note the difference in dynamic range used to display the *R*_2_* maps at the two field strengths. (**B**) Linear regression analysis of *R*_2_* measurements obtained from tumour-bearing transgenic mice imaged at both 3 and 7T revealed a significant positive correlation (*R*=0.87, *P*=0.0012). Parametric (**C**) *T*_1_ and (**D**) *R*_2_* maps acquired at 3T from representative tumour-bearing Th-*MYCN* and Th-*ALK*^*F1174L*^/Th-*MYCN* transgenic mice. Summary plots showing the quantitative data for (**E**) native *T*_1_ and (**F**) *R*_2_*. Data are individual tumour medians, and the mean±1 s.e.m., ***P<0.001.

Parametric *R*_2_* maps from a tumour arising in a Th*-MYCN* mouse acquired at both 3 and 7T are shown in Figure 3A. While anatomical *T*_2_-weighted images revealed some anticipated differences in animal positioning, it was possible to identify similar tumour positions at both field strengths. A heterogeneous distribution of *R*_2_* values was apparent across all tumours imaged at both field strengths. A statistically significant positive correlation of *R*_2_* values quantified at 3 and 7T was determined (*n*=10, *R*=0.87, *P*=0.0012, Figure 3B). Unlike the phantom data, no significant correlation in tumour *T*_1_ relaxation times measured at 3 and 7T *in vivo* was found. This may be a consequence of different methods of *T*_1_ quantification (multiple slice variable flip angle approach at 3T *vs* single slice inversion-recovery at 7T), impossibility of achieving the exact same positioning and acquiring data from the same central slice on the two imaging platforms, and the absence of any *B*_1_ correction for the data acquired at 3T.

Parametric maps of native *T*_1_ and *R*_2_* obtained at 3T from representative Th-*MYCN* and Th*-ALK*^*F1174L*^*/*Th*-MYCN* mice are shown in Figure 3C and D. A more homogeneous distribution of both *T*_1_ and *R*_2_* values was apparent in tumours arising in the Th*-ALK*^*F1174L*^*/*Th*-MYCN* mice. The quantitative data are summarised in Figure 3E and F. While there was no significant difference in native tumour *T*_1_ between the Th-*MYCN* and the Th*-ALK*^*F1174L*^*/*Th*-MYCN* mice (1098±71 ms and 1149±118 ms; *P*>0.1), *R*_2_* was significantly faster in tumours in the Th-*MYCN* mice when compared to the Th*-ALK*^*F1174L*^*/*Th*-MYCN* cohort (49.7±4 s^−1^ and 27.7±3 s^−1^; *P*=0.0007).

**Figure 4:**
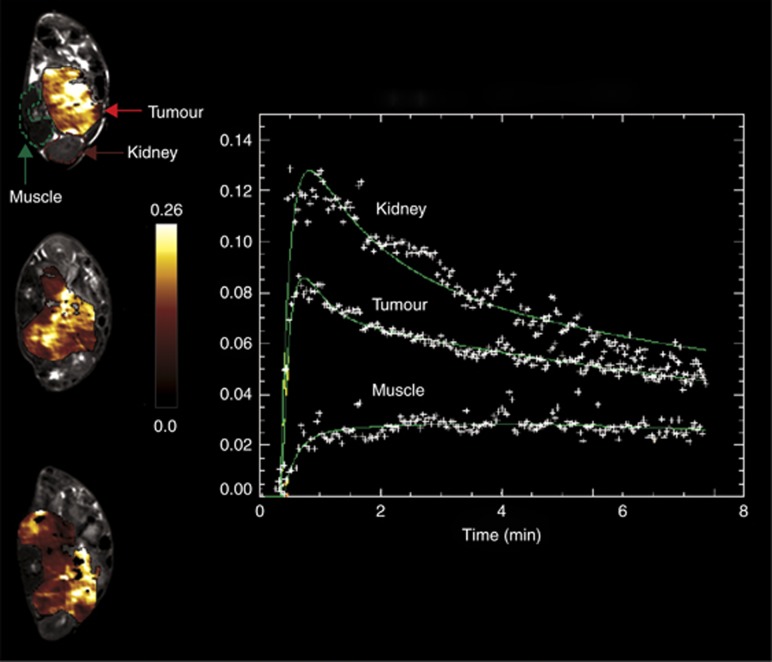
**Simultaneous DCE-MRI at 3T.** Parametric *K*^trans^ maps obtained from three tumour-bearing transgenic mice from which DCE-MRI data were simultaneously acquired using the high resolution 3-channel/3-animal MR RF coil on the 3T clinical scanner, showing a heterogeneous distribution of vascular permeability/perfusion across all three neuroblastomas. Gadolinium uptake curves obtained from one mouse for ROIs positioned over the tumour, kidney and paraspinal muscle are shown. Median *K*^trans^ values of 0.202, 0.168 and 0.114 min^−1^ were estimated from the upper, middle and lower tumour ROIs, respectively.

DCE-MRI was successfully performed on three transgenic mice simultaneously using the 3T platform, yielding data with sufficient temporal and spatial resolution to evaluate vascular biomarkers of each imaged tumour *in vivo*. Pharmacokinetic modelling was employed to derive and spatially map DCE-MRI parameters. Parametric *K*^trans^ maps obtained from each tumour-bearing mouse are shown in Figure 4, along with representative contrast agent uptake curves obtained from ROIs positioned over the tumour, kidney and paraspinal muscle. The mean of median and range values of *K*^trans^, *V*_e_ and IAUGC_60_ acquired from the three simultaneously imaged animals were 0.161 min^−1^ (0.124–0.202 min^−1^), 0.396 (0.374–0.415) and 3.28 (2.38–4.27) mM.s, respectively.

**Figure 5:**
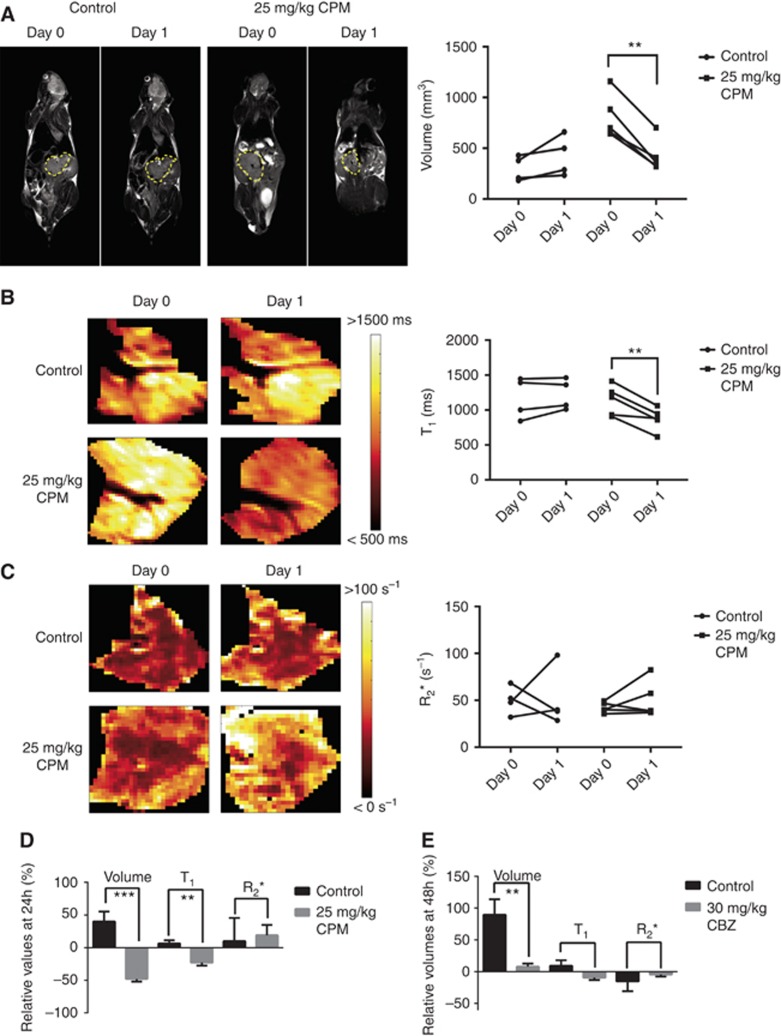
**Assessment of tumour response to CPM or CBZ treatment using the 3T platform.** (**A**) *T*_2_-weighted coronal images acquired at 3T using the high resolution 3-channel/3-animal MR RF coil from Th-*MYCN* transgenic mice bearing abdominal neuroblastomas prior to and 24 h after treatment with either saline (control) or CPM. Each tumour is delineated in yellow. Treatment with CPM resulted in a consistent and significant (**P<0.01) reduction in tumour volume. Parametric maps of tumour (**B**) native *T*_1_ and (**C**) *R*_2_* acquired at 3T from Th-*MYCN* mice prior to and 24 h after treatment with either saline (control) or CPM. While treatment with CPM elicited a significant reduction in native *T*_1_ (**P<0.01), there was no change in tumour *R*_2_*. Relative changes in tumour volume, native *T*_1_ and *R*_2_* determined from Th-*MYCN* mice treated with vehicle, or either CPM or CBZ, are shown in (**D**) and (**E**) respectively. Data shown are the individual tumour median values and the mean±1 s.e.m. (***P<0.001, **P<0.01).

Anatomical images and parametric maps acquired from representative Th-*MYCN* mice prior to and 24 h after treatment with saline or CPM are shown alongside individual tumour volume, *T*_1_ and *R*_2_* values in Figure 5A–C, and summarised in Figure 5D. Treatment with CPM elicited a significant (*P*=0.0015) reduction in tumour burden, and was associated with a significant decrease in native *T*_1_ (*P*=0.0085). Collectively this translated to a significant difference in both relative tumour volume (*P*=0.0004) and native *T*_1_ (*P*=0.0035) between the control and CPM groups (Figure 5D). There was no significant treatment-induced change in any parameter over 24 h in the control cohort. Treatment with CBZ elicited marked tumour growth delay compared to control, resulting in a significant difference in the relative tumour volume (*P*=0.0076). There was however no associated significant change in relative native *T*_1_ or *R*_2_* with CBZ, and no significant treatment-induced change in any parameter over 48 h in the control group (summarised in Figure 5E).

## Discussion

Pre-existing and new clinical MRI scanners operating between 1 and 3T, available to clinically orientated research groups, are being increasingly used for pre-clinical imaging studies in rodents (Dazai *et al*, 2004; Brockmann *et al*, 2007; Chen *et al*, 2007; Inderbitzin *et al*, 2007; Linn *et al*, 2007; Nieman *et al*, 2007; Jerome *et al*, 2016). The potential and advantages of simultaneous MRI of multiple tumour-bearing animals has been highlighted (Dazai *et al*, 2004; Li *et al*, 2005; Beuf *et al*, 2006; Wilmes *et al*, 2007). Such pre-clinical applications require the use of appropriately adapted or dedicated RF coils for small fields of view, especially when the acquisition of functional MRI data is considered. For dedicated clinical MRI research centres, the use of such a coil arrangement for performing pre-clinical studies is however more cost-effective compared to low-field benchtop MRI systems that are becoming increasingly available for routine pre-clinical investigations. The necessity to generate linear magnetic field gradients over greater volume on clinical systems limits the geometrical design and hence gradient system performance in comparison to dedicated pre-clinical systems. As a consequence, maximum gradient strengths and slew rates are typically lower, which is also often desirable in order to reduce patient peripheral nerve stimulation. It can however limit EPI-based acquisitions such as DWI, rapid or ultra-high resolution imaging.

In this study, the utility and sensitivity of anatomical and functional MRI data, acquired using a high resolution RF coil on a clinical 3T scanner, from two transgenic mouse models of neuroblastoma was evaluated. In addition, the data were cross-referenced to multiparametric data acquired from the same transgenic mice on a dedicated pre-clinical 7T system, with a particular focus on quantification of native longitudinal relaxation time *T*_1_, and transverse relaxation rate *R*_2_*, previously shown to be sensitive imaging biomarkers of treatment response and geno/phenotype respectively (Jamin *et al*, 2013, 2014).

Use of the high resolution 3-channel/3-animal RF coil on a clinical 3T platform yielded high quality *T*_2_-weighted anatomical images of up to three mice simultaneously, with sufficient resolution to accurately define and quantify the volume of neuroblastomas arising within the abdomen of the transgenic mice *in vivo*. The limited region of homogeneous field on dedicated high field pre-clinical systems usually limits the acquisition of functional imaging data from just a few representative slices across the centre of a tumour. This is not the case for clinical systems which allow functional measurements to be made across the whole tumour. In addition, the multiple coil arrangement enabling simultaneous scanning of several subjects enables high throughput ideally suited for efficient cohort screening.

In addition, acquisition of whole mouse body data enables the detection of any distant metastasis in the same imaging session, a clear advantage when compared to the imaging performed on high-field pre-clinical scanners with a more limited region of magnetic field homogeneity. In cancer research, the increasing development and use of more sophisticated orthotopic and transgenic mouse models of primary and secondary disease demands accurate non-invasive imaging methods to confirm successful engraftment/propagation and longitudinal monitoring of deep-seated tumours *in vivo* (Bielen *et al*, 2012; Jamin *et al*, 2013; Graham *et al*, 2014). The coil arrangement used herein can clearly be used to facilitate such tumour model development, provides a potential screening tool to confirm tumourigenesis, and may alleviate demand at research establishments where the availability/capacity for high-field imaging is limited.

The ability to quantify functional MRI data using the 3-channel/3-animal RF coil at 3T was also explored. The primary objective was quantification of tumour native *T*_1_ and *R*_2_*, imaging biomarkers previously shown to be sensitive to successful treatment response and haemodynamic vasculature within the Th-*MYCN* and Th*-ALK*^*F1174L*^*/*Th*-MYCN* mice, measured at 7T (Jamin *et al*, 2013, 2014). A key aim was to achieve a good compromise between sufficient resolution, SNR and scan time for quantitation of native *T*_1_ and *R*_2_*, rather than replicating sequences and parameters routinely used on the 7T system. The excellent linearity and positive correlation determined using the *T*_1_ phantom, and the strong significant correlation of tumour *R*_2_* values *in vivo,* determined across both scanners, suggests that the coil has sufficient sensitivity to acquire and accurately measure native *T*_1_ and *R*_2_* in these transgenic mouse models of neuroblastoma. As expected, the native *T*_1_ relaxation times in tumours arising within the Th-*MYCN* mice were lower at 3T than those previously reported at 7T (Sasaki *et al*, 2005; Jamin *et al*, 2013). The *R*_2_* values acquired in Th-*MYCN* and Th*-ALK*^*F1174L*^*/*Th*-MYCN* were ∼43% lower than those determined at 7T in this study, are consistent with previously published values (Jamin *et al*, 2014), and with those reported from human tumours at 3T (Hallac *et al*, 2012; Li *et al*, 2015; Bane *et al*, 2016; Panek *et al*, 2016). A ratio of 2.14 was found between tumour *R*_2_* measured at 3 and 7T (Figure 3B), which is in agreement with the expected linear increase of the deoxyhaemoglobin *R*_2_* relaxivity with magnetic field strength. An *R*_2_* ratio of 2.98 has been previously reported for pure blood at 3 and 7T (Blockley *et al*, 2008). Neuroblastoma is inherently hypervascular, hence quantitation of *R*_2_*, sensitive to paramagnetic deoxyhaemoglobin, using intrinsic susceptibility MRI is being actively investigated both pre-clinically and clinically as a case-specific imaging biomarker for this paediatric cancer. Encouragingly, the significantly slower *R*_2_* values determined at 3T in the tumours arising within the Th*-ALK*^*F1174L*^*/*Th*-MYCN* mice is wholly consistent with their impaired haemodynamic vascular phenotype relative to that in the *Th-MYCN* mice, previously established using intrinsic susceptibility MRI at 7T (Jamin *et al*, 2014), supporting the concept of ‘MRI genotyping’ *in vivo* (Nieman *et al*, 2007).

Previous studies have described the acquisition of contrast-enhanced MRI data from tumour-bearing rodents (individually or in pairs) on clinical scanners, used to inform on either treatment stratification or treatment response (Li *et al*, 2005; Muruganandham *et al*, 2006; Wilmes *et al*, 2007; Egeland *et al*, 2009; Hompland *et al*, 2012). In our study we demonstrated the feasibility of performing DCE-MRI simultaneously on three mice using the 3-channel/3-animal RF coil at 3T, enabling the successful pharmacokinetic analysis of Gd-contrast uptake curves in tumours and surrounding organs. The use of volume coils with geometries suitable for small rodents enabled high SNR DCE-MRI measurements with sufficient resolution and coverage necessary for pre-clinical tumour imaging. Furthermore, images are fully compatible with processing tools developed for clinical diagnosis and therapy planning software. This enables evaluation of methods used for multi-parametric and sub-regional tumour analysis, signal modelling (i.e. DCE and DWI models), or methodological MR imaging development. In our study, clinically developed software (MRIW, ADEPT) was used to calculate parametric *T*_1_ and *R*_2_* maps, and to perform pharmacokinetic modelling of the DCE-MRI data.

Finally, the utility of the 3-channel/3-animal RF coil for performing pre-clinical interventional trials at 3T was investigated by assessing the anatomical and functional MRI response of tumours arising in Th-*MYCN* transgenic mice to CPM, the current standard treatment of care for children with high-risk neuroblastoma, or CBZ, currently in clinical trials for the treatment of neuroblastoma. Treatment with CPM resulted in a significant decrease in both tumour volume and native *T*_1_, consistent with previous results acquired at 7T (Jamin *et al*, 2013). Significant reductions in tumour native *T*_1_ following successful treatment with a range of therapeutic regimes have been reported both pre-clinically and clinically, hypothesised to be a consequence of the release of proteins and paramagnetic ions into the extracellular space (O’Connor *et al*, 2009; McSheehy *et al*, 2010; Jamin *et al*, 2013). Treatment with CBZ elicited a cytolentic response only. Collectively these data demonstrate the ability to reveal differential tumour response to treatment using this coil arrangement and multiparametric MRI.

There are several methodological and logistical limitations to performing pre-clinical investigations on clinical MRI systems. A reliable and repeatable shim over a small volume of interest poses a challenge, especially if EPI-based techniques (i.e. diffusion weighted imaging) and spectroscopy are considered. This can contribute to a decrease of *R*_2_* linearity measured at both fields. However, differences in the positioning of the imaged volumes, and the relatively high tumour heterogeneity apparent in Th-*MYCN* tumours, should also be considered. There are practical considerations in positioning lines for the remote, simultaneous administration of contrast agents and delivery of gaseous anaesthetic from the scanner control room, hence the use of injectable anaesthesia in these studies. The utility of this coil arrangement for imaging tumours arising in different anatomical sites within other transgenic and orthotopic mouse models of cancer needs to be established. Finally, significant effort must be made to conform to institutional local rules and minimise any health risk associated with cross-contamination, and to recognise the limited access to clinical scanners during normal hospital operating hours.

In conclusion, we have demonstrated the utility and sensitivity of a high resolution 3-channel/3-animal MR RF coil for performing informative anatomical and functional MRI studies in transgenic mouse models of neuroblastoma on a clinical 3T system. In this way, simultaneous data acquisition in multiple tumour-bearing animals can provide high efficiency/throughput for both phenotypic characterisation and trials of novel therapeutics, with additional mechanistic insight provided by multiparametric MRI, improving the accuracy of pre-clinical data and facilitating the introduction of functional MRI biomarkers into aligned imaging-embedded clinical trials.

## Figures and Tables

**Table 1:** Longitudinal relaxation times quantified from the gadolinium phantoms at 3 and 7T

**[Gd]**	**7T**	**3T**
Saline	2358±21	1608±223
30 nm	1811±34	1238±84
40 nm	1605±14	1002±83
50 nm	1499±15	950±132
100 nm	1227±178	704±7
500 nm	400±4	241±3

T_1_ values (ms) were determined from solutions of increasing concentration of gadopentate measured four times on the clinical 3T and pre-clinical 7T MRI scanners.

Data are mean of the medians±1 s.e.m.
